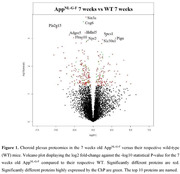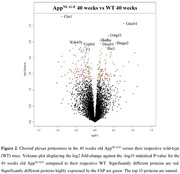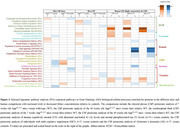# Involvement of the choroid plexus in Alzheimer’s disease pathophysiology: A mouse and human proteomic study

**DOI:** 10.1002/alz.090722

**Published:** 2025-01-03

**Authors:** Aurore Delvenne, Charysse Vandendriessche, Johan Gobom, Marlies Burgelman, Pieter Dujardin, Clint De Nolf, Betty M. Tijms, Suzanne E. Schindler, Frans R.J. Verhey, Inez H.G.B. Ramakers, Henrik Zetterberg, Pieter Jelle Visser, Roosmarijn E Vandenbroucke, Stephanie J. B. Vos, EMIF‐AD MBD study group

**Affiliations:** ^1^ Alzheimer Center Limburg, School for Mental Health and Neuroscience, Maastricht University, Maastricht Netherlands; ^2^ VIB‐UGent Center for Inflammation Research, Ghent Belgium; ^3^ Department of Biomedical Molecular Biology, Ghent University, Ghent Belgium; ^4^ Clinical Neurochemistry Laboratory, Sahlgrenska University Hospital, Gothenburg, VGR Sweden; ^5^ Department of Psychiatry and Neurochemistry, Institute of Neuroscience and Physiology, The Sahlgrenska Academy, University of Gothenburg, Mölndal Sweden; ^6^ VIB Center for Inflammation Research, Ghent Belgium; ^7^ Ghent University, Ghent Belgium; ^8^ Alzheimer Center Amsterdam, Neurology, Vrije Universiteit Amsterdam, Amsterdam UMC location VUmc, Amsterdam Netherlands; ^9^ Washington University in St. Louis School of Medicine, St. Louis, MO USA; ^10^ Knight Alzheimer Disease Research Center, St. Louis, MO USA; ^11^ Maastricht University, Alzheimer Center Limburg, School for Mental Health and Neuroscience, Maastricht Netherlands; ^12^ UCL Institute of Neurology Queen Square London UK, London United Kingdom; ^13^ Institute of Neuroscience and Physiology, Department of Psychiatry and Neurochemistry, The Sahlgrenska Academy, University of Gothenburg, Mölndal, Gothenburg Sweden; ^14^ Alzheimer Center and Department of Neurology, Amsterdam Neuroscience Campus, VU University Medical Center, Amsterdam Netherlands

## Abstract

**Background:**

Structural and functional changes of the choroid plexus (ChP) have been reported in Alzheimer’s disease (AD). Nonetheless, the role of the ChP in the pathogenesis of AD remains largely unknown. We aim to unravel the relationship between ChP functioning and core AD pathogenesis using a unique proteomic approach in mice and humans.

**Method:**

We used an App knock‐in mouse model, App^NL‐G‐F^, with amyloid pathology to study the association between AD brain pathology and protein changes in mouse ChP tissue and CSF, using liquid chromatography mass spectrometry. Mouse proteomes were investigated at 7 weeks old (n = 5) and at 40 weeks old (n = 5). Results were compared to human AD CSF proteomic data (n = 496; from EMIF‐AD MBD, Knight ADRC, and Maastricht BB‐ACL studies) to identify key proteins and pathways associated with ChP changes in AD.

**Result:**

Compared to wild‐type (WT) mice, App^NL‐G‐F^ mice aged 7 or 40 weeks old had significant differences in some protein levels in ChP tissue (Figure 1 and 2). At both ages, ChP tissue proteomic changes were related to pathways associated with epithelial cells, mitochondrion, protein modification, extracellular matrix (ECM) and lipids. Pathways related to lysosomal function, endocytosis, protein formation, actin, and complement were uniquely dysregulated at 7 weeks, while pathways associated with nervous system, immune system, protein degradation and vascular system were uniquely dysregulated at 40 weeks. Mouse CSF proteomics showed changes in similar pathways as seen in ChP tissue proteomics (Figure 3). Proteomic analyses were also performed in non‐demented human participants with abnormal amyloid but normal tau levels. Among proteins that were up‐regulated in CSF of those participants compared to controls, a significant number were predominantly expressed in the ChP (38 to 56% of the up‐regulated proteins). The dysregulated proteins with a predominant expression in the ChP in those individuals were related to similar pathways as seen in App^NL‐G‐F^ mice ChP tissue and CSF proteomics (Figure 3).

**Conclusion:**

ChP changes are associated with amyloid pathology. Key pathways involved in ChP dysfunction in AD are associated with the ECM, lysosomal function, lipids, protein processing, complement, vascular system and mitochondrion. The ChP could become a novel promising target for AD treatment.